# Rare Variants of Immune-Related Genes Increase Susceptibility to Autoimmune Encephalitis: An Association Study

**DOI:** 10.3390/neurolint17120199

**Published:** 2025-12-08

**Authors:** Chih-Hsiang Lin, Shiau-Ching Chen, Chen-Jui Ho, Che-Wei Hsu, Shih-Ying Chen, Yan-Ting Lu, Meng-Han Tsai

**Affiliations:** 1 Department of Neurology, Kaohsiung Chang Gung Memorial Hospital, Chang Gung University College of Medicine, Kaohsiung 833401, Taiwan; thsign@cgmh.org.tw (C.-H.L.);; 2 School of Medicine, College of Medicine, Chang Gung University, Taoyuan 333323, Taiwan; 3 Department of Neurology, Kaohsiung Municipal Feng-Shan Hospital (Under Management of Chang Gung Medical Foundation), Chang Gung University College of Medicine, Kaohsiung 833401, Taiwan; 4 Department of Medical Research, Kaohsiung Chang Gung Memorial Hospital, Kaohsiung 833401, Taiwan; 5 Doctoral Program of Clinical and Experimental Medicine, College of Medicine, National Sun-Yet Sen University, Kaohsiung 833401, Taiwan

**Keywords:** autoimmune encephalitis, immunological genes, rare deleterious variants

## Abstract

Introduction: Autoimmune encephalitis (AE) is a neurological disorder caused by immune responses targeting neuron-surface or synaptic proteins. While its immunological mechanisms have been studied, the genetic underpinnings remain unclear. This study investigates whether rare deleterious variants (RDVs) in immunological genes contribute to AE susceptibility. Method: We enrolled 36 patients with AE and 407 healthy controls without autoimmune diseases. Whole-exome sequencing was performed to identify RDVs, including start-loss, stop-gain, frameshift, splice-site variants, and deleterious missense mutations. We analyzed the distribution of RDVs in an immunological gene set and its subsets. A burden test was used to identify genes significantly associated with AE. Results: Overall, RDVs in the full immunological gene set did not differ between AE patients and controls. However, the T cell receptor signaling pathway subset showed a significantly higher RDV burden in AE patients. Within this pathway, *PDK1* was significantly associated with AE. Two additional genes, *CAT* and *MIA*, also showed strong associations, although their broader gene subset, cytokines, did not display differential RDV distribution. Discussion: Our findings suggest that RDVs in specific immunological pathways, particularly the T cell receptor signaling pathway, may play a role in AE pathogenesis. The significant associations of *PDK1*, *CAT*, and *MIA* with AE highlight potential genetic contributors to the disease. Further functional studies are necessary to validate these associations and explore their biological relevance, potentially paving the way for improved understanding and future therapeutic targets in AE.

## 1. Introduction

Autoimmune encephalitis (AE) is caused by the production of autoantibodies against the neuron-surface or synaptic proteins [1] that results in a wide spectrum of neuropsychiatric manifestations, including acute psychosis, cognitive impairment, seizures, and/or involuntary movement [2]. If not treated immediately, permanent neurological damage may lead to disability or death. However, the diagnosis of AE is not straightforward and depends heavily on the clinical presentation based on experts’ opinions [3]. Clinical examinations, including cerebrospinal fluid (CSF) analysis, magnetic resonance imaging (MRI), genetic testing, and electroencephalography (EEG) are used to differentiate between other diseases with similar presentations. These conditions may include central nervous system (CNS) infections, CNS inflammatory diseases like CNS vasculitis, Susac syndrome, or Behcet’s disease, non-inflammatory CNS diseases such as Creutzfeldt-Jakob disease, cryptogenic new-onset refractory status epilepticus (NORSE), mitochondrial disease, psychosis due to psychiatric disorders, primary brain tumors like primary CNS lymphoma, and other metabolic encephalopathies [4,5,6]. A lack of understanding of the underlying pathogenesis hinders the discovery of biomarkers for diagnosing AE and developing effective treatments to halt the aberrant immune responses.

The precise mechanism underlying the production of neuronal autoantibodies remains elusive. One theory suggests that CNS antigens may activate naïve B lymphocytes, leading to the production of autoantibodies [7]. Alternatively, it is proposed that external antigens resembling CNS antigens, such as viruses, bacteria, or tumors, may trigger immune system activation [8,9]. Clinical observations have found that following herpes simplex virus (HSV) infection, anti-N-methyl-d-aspartate receptor (NMDAR) encephalitis may develop in susceptible individuals [10]. Tumors have been implicated in producing autoantibodies by expressing neuronal antigens, leading to various forms of AE, including anti-NMDAR encephalitis [11] or leucine-rich glioma-inactivated 1 (LGI1) antibody encephalitis [12]. These observations suggest that patients susceptible to AE may have compromised immune homeostasis, making them more prone to unwanted immunological reactions upon exposure to various antigens.

Numerous association studies have implicated genetic polymorphisms in contributing to susceptibility to AE, mainly limited to the human leukocyte antigen [13,14] and interleukin genes [15]. To assess the risk of developing AE, we utilized whole-exome sequencing (WES) technology, which can sequence all coding genes simultaneously. We hypothesize that genetic variants in immune-related genes may increase susceptibility to AE, highlighting the importance of identifying such genetic markers to improve risk assessment and deepen our understanding of AE pathogenesis.

## 2. Material and Methods

### 2.1. Ascertainment of Subjects

The patient group was selected from our previous AE studies [16,17,18], while the control group, comprising healthy individuals without autoimmune diseases, was drawn from our earlier genetics studies and sequenced using the same WES platform. All subjects included in the study are Taiwanese adults aged over 20 years, all of whom underwent WES. The studies were approved by the Chang Gung Medical Foundation Institutional Review Board. Written informed consents of all study participants were obtained.

Patients included in the study should meet the diagnostic algorithm as per the experts’ consensus [3]. Firstly, they must satisfy the diagnostic criteria for possible AE (Supplementary Table S1). Following this, only those who met the diagnostic criteria for either definite autoimmune limbic encephalitis (Supplementary Table S2) or anti-NMDA receptor encephalitis (Supplementary Table S3) were included in the study. Patients with acute disseminated encephalomyelitis, Bickerstaff’s brainstem encephalitis, and Hashimoto’s encephalopathy were excluded. All patients included in the study were tested for the presence of antibodies against neuronal surface antigens in the serum and CSF, including anti-NMDA, anti-AMPA, anti-LGI1, anti-CASPR2, anti-DPPX, and anti-GABAB receptors, using a commercial kit (Autoimmune Encephalitis Mosaic 6 Assay, EUROIMMUN, Lübeck, Germany). Patients who tested negative for the commercial kit were included only if they fulfilled the diagnostic criteria of probable antibody-negative AE (Supplementary Table S4) [6] and were responsive to immunotherapy.

All patients received routine clinical surveys, including blood biochemistry, CSF study, EEG, and brain MRI to rule out diseases that might mimic AE, such as CNS infection, other inflammatory or non-inflammatory diseases of the CNS, brain tumor, or metabolic encephalopathies. Markers of systemic autoimmune disease, such as anti-nuclear antibodies, anti-dsDNA, anti-extranuclear antigens, or antiphospholipid antibodies, were tested to rule out rheumatologic disorders that may involve the CNS [19]. Computed tomography of the chest and/or abdomen was performed to discover underlying tumors.

### 2.2. Whole-Exome Sequencing and Bioinformatics Analysis

Genomic DNA was extracted from peripheral blood mononuclear cells for germline sequencing. All AE patients and controls underwent WES using the MGI DNBSEQ-G400 platform. To minimize false-positive variant calls, we applied the following filtering criteria: QUAL < 10, MQ < 30, FS > 60, RPRS < −8, MQRS < −12.5, DP < 10, GQ < 20, AD/DP < 0.25 (for heterozygous variants). Genetic variants were annotated using the Ensembl Variant Effect Predictor (VEP) [20]. We further filtered the variants to include only those with a minor allele frequency (MAF) < 0.01 in the Genome Aggregation Database (gnomAD) [21] and restricted them to variants with an MAF < 0.01 within our case–control cohort.

We aimed to find the rare deleterious variants (RDVs) associated with AE. RDV denotes a subset of variations within the sequence data that meet criteria designed to enrich for pathogenic variants based on population allele frequency and predicted functional effect [22]. We specifically focused on missense, start-loss, stop-gain, frameshift, and splice variants, categorizing them into three groups. The first group, loss-of-function (LoF) variants, includes start-loss, stop-gain, frameshift, and splice variants. Splice variants were further filtered using SpliceAI [23], with a predicted score threshold of >0.8 for inclusion in the analysis. The second group, deleterious missense (dMis) variants, comprises missense variants predicted to be deleterious by all three in silico tools: REVEL [24] with a score > 0.9, AlphaMissense [25] with a score > 0.6, and CADD [26] with a score > 15. The third group, total RDVs (tRDV), includes all variants meeting the criteria for either dMis or LoF variants.

The Mann–Whitney U test was used to compare the rank distributions of RDVs between AE patients and controls, with the results interpreted based on the median number of RDVs across the immunological gene set and its subsets. The immunological gene set and subsets are obtained from the Immunology Database and Analysis Portal (ImmPort) system (https://www.immport.org/shared/genelists, accessed on 7 August 2022) [27], which comprises 1793 known immunological genes categorized into 17 subsets. A burden test was performed using RVtest software (version 2.1.0) to investigate the association between RDVs and AE [28]. The test employed the combined multivariate and collapsing (CMC) method for analysis [29]. In summary, RDVs within a gene were aggregated into a single binary variable, coded as 1 if RDVs were present and 0 if absent. We then tested the difference in the proportion of individuals with RDVs between the case and control groups. The selection of the CMC method was based on its direct correspondence to our biological hypothesis. We specifically targeted variants with a high predicted functional impact (LoF and dMis) and assumed these rare variants would increase the risk for AE. The CMC method, as a collapsing approach, is optimally powered to detect a burden of rare deleterious variants when they act in the same direction, which aligns perfectly with our hypothesis. While Kernel methods (like SKAT) are more robust to mixed risk/protective effects, their power is attenuated when the risk is predominantly unidirectional and restricted to highly damaging variants, as is the case in our study. Therefore, CMC provided the most appropriate and statistically powerful test for our specific rare variant hypothesis.

### 2.3. Structural Analysis

To predict the impact of the genetic variant caused by the RDV, we performed computer simulations to evaluate how structural changes might alter the interaction between the protein and its ligand or interacting partners. The protein structure of the gene harboring the RDV was obtained from the AlphaFold Protein Structure database (https://alphafold.ebi.ac.uk/, accessed on 10 August 2024) [30] and modified to incorporate the genetic variant using Swiss-PdbViewer (version 4.1.0) (https://spdbv.unil.ch/, accessed on 10 August 2024) [31]. MolModa (version 1.0.1) [32], a browser-based platform for molecular docking (https://durrantlab.pitt.edu/molmoda/, accessed on 10 August 2024), was used to predict differences in docking poses and binding free energies (kcal/mol) between proteins and their ligands, with and without the genetic variant. This tool supports standard molecular docking workflows and enables docking simulations between proteins and small-molecule compounds of interest. For proteins involved in interactions with other proteins, we used ClusPro (version 2.0) (https://cluspro.bu.edu/publications.php, accessed on 10 August 2024) [33] to simulate the impact of mutations on these interactions. ClusPro is a web-based platform that employs rigid-body docking to model protein–protein binding. We compared the interaction patterns of wild-type and mutant proteins to evaluate whether the mutation induced any structural or binding alterations.

## 3. Results

A total of 36 patients with AE and 407 controls were collected. Our cohort consisted of 252 females and 191 males with a median age of 32.0 (IQR = 23.0–41.0). There was no statistically significant difference in the distribution of sex or age between the two groups. Among the case group, 12 patients had anti-NMDAR encephalitis, one had anti-GABAB receptor encephalitis, one had anti-LGI1 antibody encephalitis, and 22 had probable antibody-negative AE. The demographic characteristics of our case group are shown in Table 1.

### 3.1. Distribution of Rare Deleterious Variants (RDVs) Between Patients and Controls

Among the immunological gene set, patients with AE exhibited 20 LoF variants, 2 dMis variants, and 22 tRDVs, compared to 173 LoF variants, 21 dMis variants, and 194 tRDVs in the control group (Supplementary Table S5). The average number of RDVs per person was numerically higher in AE patients than in controls across all three RDV types: 0.56 versus 0.43 for LoF, 0.06 versus 0.05 for dMis, and 0.61 versus 0.48 for tRDV. However, since the distribution was not normally distributed, the Mann–Whitney U test was applied to compare the rank distribution of RDVs, which revealed no statistically significant differences in the median number of RDVs (location parameter) between AE patients and controls for any of the three RDV types. Among the 17 immunological gene subsets, only the T cell receptor signaling pathway showed a significantly higher distribution of RDVs in patients with AE compared to controls for both LoF variants and tRDVs (*p* = 1.62 × 10^−4^ and 2.04 × 10^−5^, respectively), but not for dMis variants (*p* = 0.772).

### 3.2. Burden Analysis of Immunological Genes

**Figure 1:**
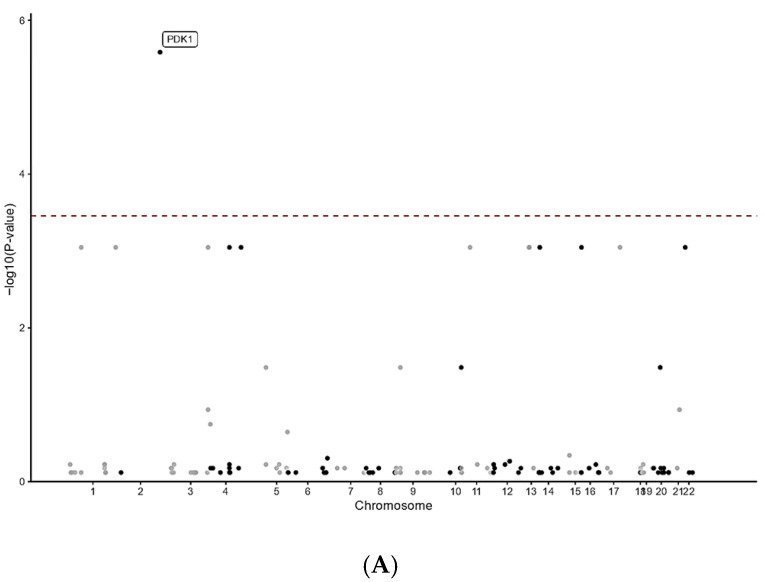

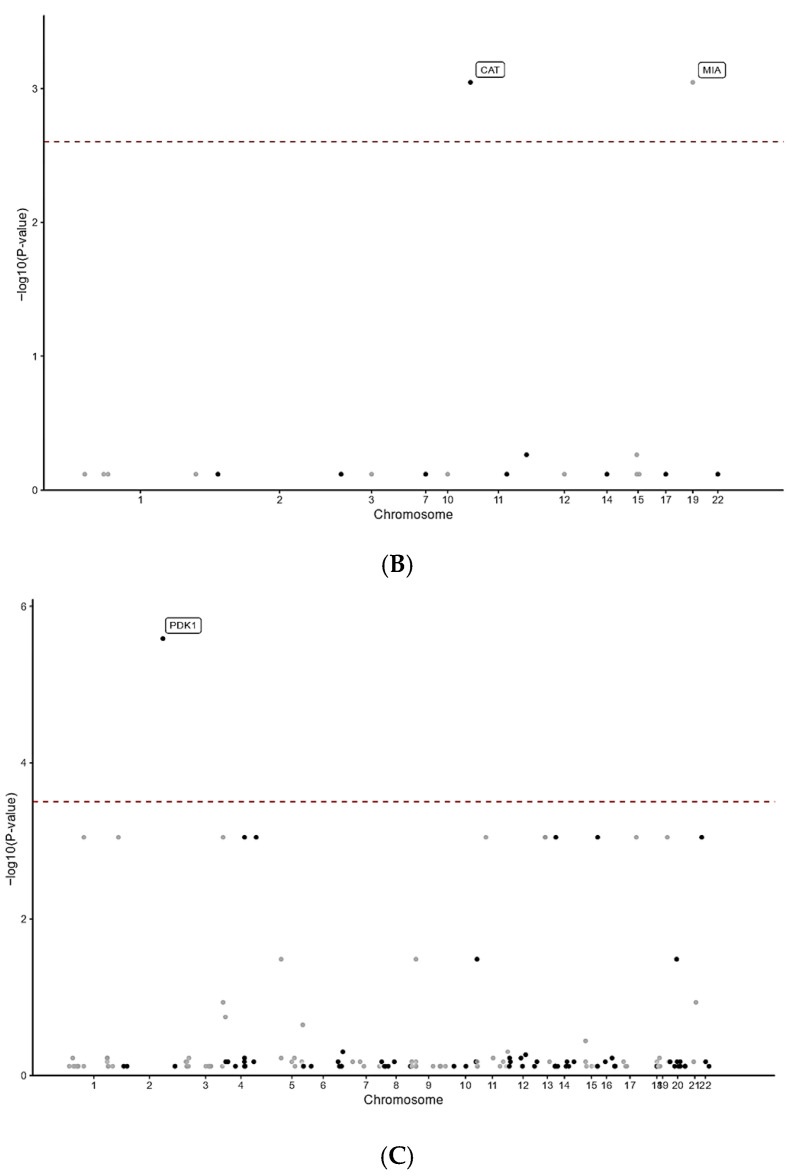
Manhattan plots for different types of rare deleterious variants, with a red line indicating the significance threshold. (**A**): Manhattan plots for loss of function variants. (**B**): Manhattan plots for deleterious missense variants. (**C**): Manhattan plots for total rare deleterious variants.

The burden of immunological gene RDVs in AE patients was compared to that in controls using CMC analysis. For LoF variants, 150 genes were tested, and a *p*-value less than 3.33 × 10^−4^ after Bonferroni correction was required to achieve statistical significance. Variants in *PDK1* (*p* = 1.88 × 10^−6^) were significantly associated with AE patients (Figure 1A). For dMis variants, among the 20 genes tested, variants in *CAT* (*p* = 7.62 × 10^−4^) and *MIA* (*p* = 7.62 × 10^−4^) surpassed the significance threshold (*p*-value < 2.5 × 10^−3^ after Bonferroni correction), showing an association with AE patients (Figure 1B). For tRDVs, among the 165 genes tested, those with *p*-values less than 3.03 × 10^−4^ after Bonferroni correction identified variants in *PDK1* (*p* = 1.88 × 10^−6^) as significantly associated with AE patients (Figure 1C).

### 3.3. Structure Analysis of the Affected Protein

**Figure 2:**
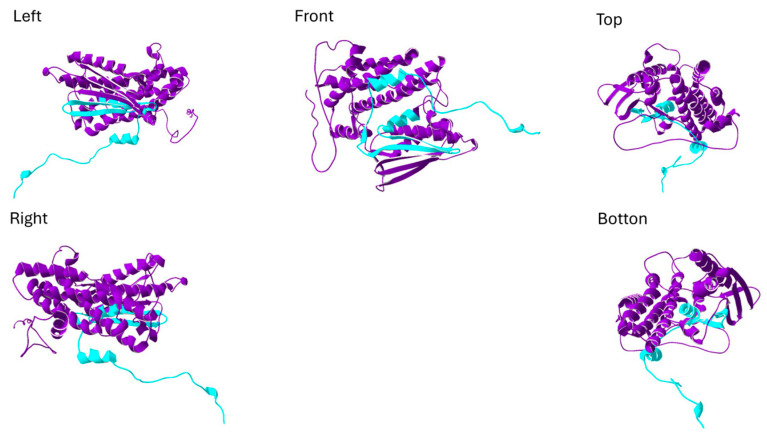
The structure of phosphoinositide-dependent kinase-1 (PDK1) is shown from five different perspectives: left, right, front, top, and bottom. The purple region represents the truncated protein fragment resulting from the PDK1:c.1072G>T, p.Gly358* mutation, while the cyan region highlights the structural loss associated with this mutation. The figure is created by SwissPdb Viewer [31].

The identified RDV in *PDK1* was *PDK1*:c.1072G>T, p.Gly358*, which is a stop-gained mutation. It was presented in two patients with probable antibody-negative AE but not in the control group. The *PDK1* gene belongs to the T cell receptor signaling pathway, consistent with our finding that AE patients harbor more RDVs in this immunological subset. Phosphoinositide-dependent kinase-1 (PDK1), encoded by *PDK1,* contains an N-terminal serine-threonine kinase domain and a C-terminal pleckstrin homology (PH) domain [34]. *PDK1*:c.1072G>T leads to a truncated protein (Figure 2) that results in the loss of the C-terminal PH domain, while the kinase domain, located between positions 80 to 342, may not be interrupted. The C-terminal PH domain of the PDK1 is critical for cellular signaling, as it binds specific phosphoinositides to facilitate the proper localization and activation of PDK1 substrates, such as AKT. These interactions are essential for regulating downstream processes, including cell growth, proliferation, and survival [35].

The RDVs in *CAT* and *MIA* were *CAT*:c.1060C>T, p.Arg354Cys and *MIA*:c.302G>A, p.Gly101Asp, respectively, both resulting in missense mutations. Each RDV was identified in a different patient with probable antibody-negative AE, and they were not the same patients harboring RDVs in *PDK1*. *CAT* and *MIA* belong to the cytokine gene subset; however, the prevalence of RDVs in this subset was not statistically different between the patient and control groups.

**Figure 3:**
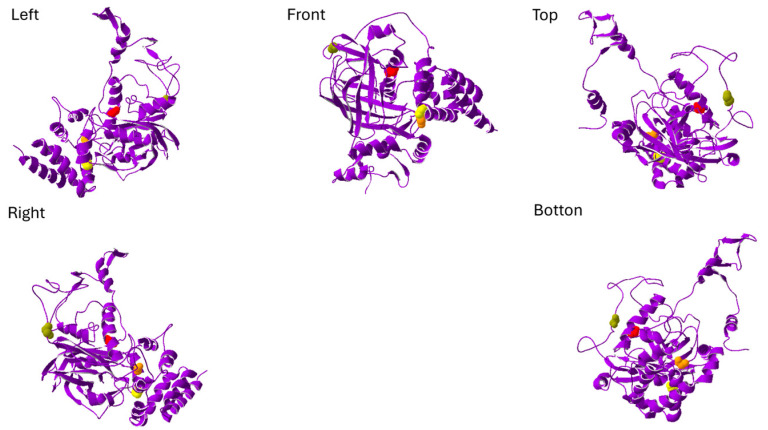
The structure of catalase is shown from five different perspectives: left, right, front, top, and bottom. The key residues involved in catalytic activity are highlighted—histidine 128 as an orange sphere, asparagine 201 as a yellow sphere, and tyrosine 415 as a green sphere. The red sphere marks the site of the amino acid substitution caused by the CAT:c.1060C>T mutation. The figure is created by SwissPdb Viewer [31].

**Figure 4:**
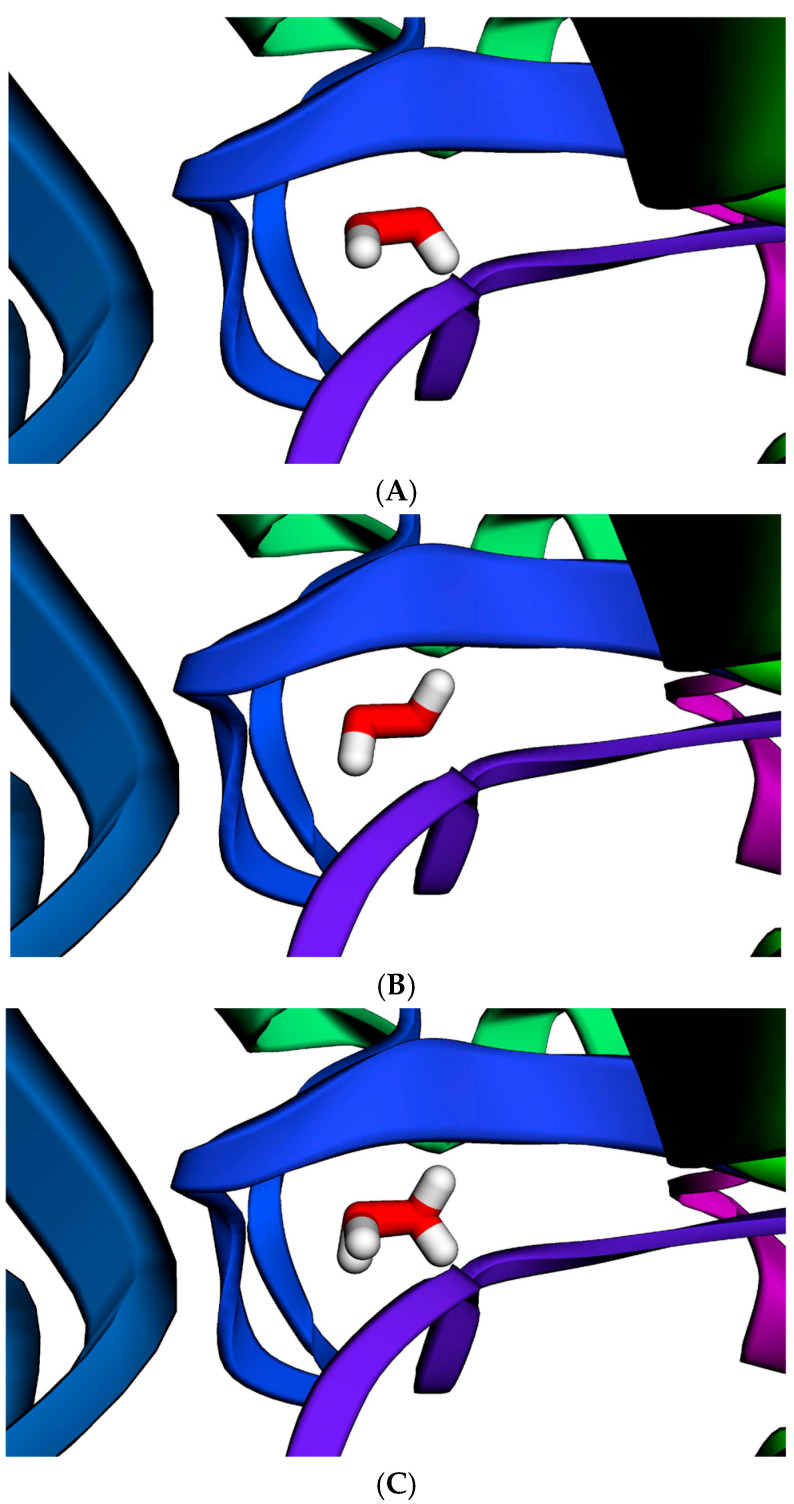
Predicted interaction of hydrogen peroxide (shown as red and white sticks) with catalase, modeled using MolModa [32]. (**A**). Hydrogen peroxide interacts with wild-type catalase between His128, Asn201, and Tyr415. (**B**). Hydrogen peroxide interacts with catalase carrying the CAT:c.1060C>T (p.Arg354Cys) mutation within the same region. (**C**). Superimposition of panels A and B demonstrates a shifted binding pose of hydrogen peroxide in the mutant structure.

The *CAT*:c.1060C>T variant results in the substitution of arginine with cysteine at position 354 (p.Arg354Cys) (Figure 3). *CAT* encodes catalase (CAT), an antioxidant enzyme that converts hydrogen peroxide into water and oxygen to protect cells from oxidative damage [36]. This variant is located within the β-barrel domain of catalase, which stabilizes the heme group essential for its catalytic function [37]. The catalytic site of catalase is formed by histidine 128, asparagine 201, and tyrosine 415, surrounding the heme pocket [37]. To evaluate the functional impact of the p.Arg354Cys mutation, molecular docking simulations were performed using MolModa [32] to model hydrogen peroxide binding. The docking scores were comparable between the wild-type and mutant catalase proteins (−2.94 kcal/mol and −2.96 kcal/mol, respectively). Although hydrogen peroxide docked to a similar region in both protein structures (Figure 4), a noticeable shift in its binding orientation was observed, suggesting that the p.Arg354Cys substitution may subtly influence substrate positioning and potentially affect enzymatic activity.

**Figure 5:**
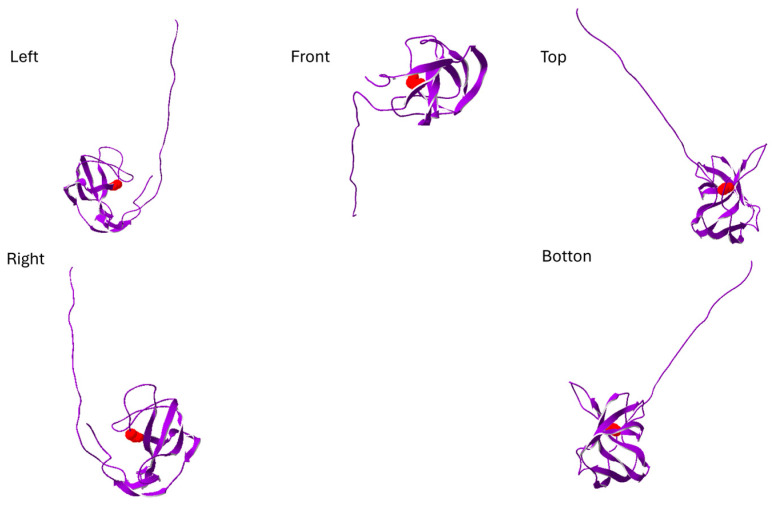
The structure of melanoma-inhibitory activity (MIA) protein is shown from five different perspectives: left, right, front, top, and bottom. The red sphere highlights the amino acid change resulting from the *MIA*:c.302G>A mutation. The figure is created by SwissPdb Viewer [31].

**Figure 6:**
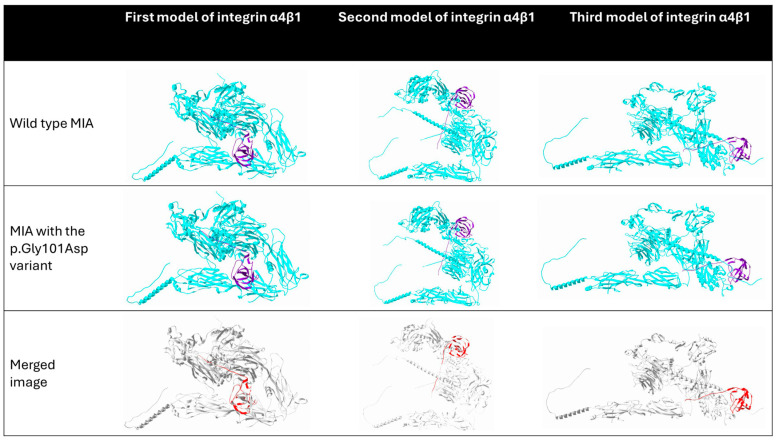
The predicted interaction between the integrin α4β1 heterodimer and the melanoma-inhibitory activity (MIA) protein, in both wild-type and p.Gly101Asp variant forms, is illustrated across three structural models of integrin α4β1. In each model, the integrin α4β1 heterodimer is shown in cyan, while the MIA protein—either wild-type or variant—is shown in purple. The bottom row presents merged images of the wild-type and variant MIA structures aligned with the same integrin model; red regions indicate areas of structural misalignment between the two forms of MIA, highlighting subtle positional shifts caused by the p.Gly101Asp variant. The figure is created by SwissPdb Viewer [31].

The *MIA*:c.302G>A variant results in the substitution of glycine with aspartic acid at position 101 (p.Gly101Asp) (Figure 5). *MIA* encodes the melanoma-inhibitory activity (MIA) protein, which binds to the integrin α4β1 [38], and we used ClusPro [33] to investigate how the identified variant might alter the binding interface or affinity between MIA and integrin α4β1. Currently, there is no publicly available structure specifically for the integrin α4β1 heterodimer. Therefore, we used ClusPro to predict the structure of the integrin α4β1 complex and selected the three best-fitting models. These integrin models were then used to evaluate the interaction between integrin α4β1 and the MIA protein in both its wild-type and variant (p.Gly101Asp) forms (Figure 6). The predicted models suggest that the variant induces only minimal changes in the interaction between integrin α4β1 and the MIA protein, with a slight positional shift in the MIA protein observed due to the genetic variant.

## 4. Discussion

In our cohort, the distribution of RDVs in the immunological gene set did not differ significantly between patients with AE and the control group. However, the T cell receptor signaling pathway, a subset of immunological genes, exhibited a significantly higher enrichment of RDVs in patients with AE compared to controls. Additionally, the burden test of immunological genes revealed a statistically significantly higher prevalence of RDVs in *PDK1*, *CAT*, and *MIA* in the case group compared to the control group. Notably, *PDK1*—a key component of the T cell receptor signaling pathway—demonstrated consistent significance across our analyses. It was not only highlighted in the burden test but also belonged to the immunological subset, which showed an increased prevalence of RDVs in patients with AE.

PDK1 is a key downstream effector of the phosphatidylinositol-3 kinase (PI3K)-Akt signaling pathway [39], which regulates immune responses in T and B cells through multiple signaling cascades [39,40]. The activation of P13K induces phosphorylation of phosphatidylinositol-(4,5)-bisphosphate (PIP2) into phosphatidylinositol-(3,4,5)-trisphosphate (PIP3) [41]. The PH domain of PDK1 and Akt then interacts with PIP3, leading to their recruitment to the plasma membrane, where PDK1 phosphorylates Akt to continue phosphorylation of downstream targets [42]. The PI3K-Akt signaling pathway plays a crucial role in adaptive immunity by allowing T cells to exit quiescence for a rapid recall immune response [39]. Deletion of *PDK1* blocks T cell differentiation [43], while its deficiency impairs B cell differentiation, prevents IgM^+^ cell generation, and increases susceptibility to cell death [44]. Currently, no functional studies have investigated the effects of the *PDK1* p.Gly358* variant. However, we hypothesize that this mutation may result in the loss of the PH domain, thereby impairing the protein’s recruitment to the plasma membrane while preserving its kinase activity. Supporting this hypothesis, a mouse study of the PDK1 Lys465Glu mutation, which disrupts the PH domain’s interaction with PIP3 and prevents membrane localization, demonstrated a significant reduction in Akt activation and downstream signaling, despite retaining partial PDK1 activity [45]. Consequently, the PDK1 p.Gly358* variant may impair immune signaling and disrupt homeostasis. Given PDK1’s critical role in adaptive immunity and the reduced PI3K-Akt pathway activity associated with this variant, it may contribute to abnormal T cell differentiation and a skewed immune response, potentially promoting autoimmune mechanisms involved in the pathogenesis of AE.

In addition to lymphocytes, monocytes/macrophages are also influenced by PDK1 [46]. The PI3K-Akt signal pathway inactivates the glycogen synthase kinase 3 (GSK3). GSK3 mediates the balance of inflammatory responses by controlling pro- and anti-inflammatory cytokine production [47]. With the inactivation of GSK3 in monocytes, the release of proinflammatory cytokines—IL-6, TNF-α, IL-12, and IFN-γ—is reduced, while the anti-inflammatory cytokine IL-10 is increased [48]. Reduced PI3K-Akt activity, due to the PDK1 p.Gly358* mutation, may impair the inactivation of GSK3, which could shift the immune response toward a proinflammatory phenotype. This disruption in immune homeostasis could contribute to autoimmunity and the development of AE.

CAT converts hydrogen peroxide into water and oxygen, protecting cells from oxidative damage [36]. Although hydrogen peroxide is a reactive molecule that must be tightly regulated to prevent cellular damage, it also functions as a key signaling molecule in the innate immune response [49]. At high concentrations, hydrogen peroxide promotes macrophage polarization toward a proinflammatory phenotype [50] and enhances neutrophil activation [51]. Our simulation showed that the binding pose of hydrogen peroxide at the catalytic site was altered by the p.Arg354Cys substitution. Although functional studies on this specific variant are not yet available, the mutation may affect hydrogen peroxide levels and, consequently, alter immune responses.

MIA is highly expressed in malignant melanoma and plays a key role in promoting tumor invasion and metastasis [52]. MIA can bind with integrin α4β1 [38], which governs the infiltration of T cells into tumor tissue [53]. An in vitro study suggested that MIA is capable of masking the expression of integrin α4β1 and suppressing the anti-tumor immune response [54]. Although MIA is classified as an immunological cytokine due to its ability to interact with integrins, there is currently no evidence linking MIA to autoimmune diseases. Given that MIA is predominantly expressed in malignant melanoma, its relationship with AE remains unclear. Our simulation further suggests that the identified variant induces only a subtle alteration in the interaction between MIA and integrin α4β1. Therefore, the observed association between MIA and AE should be interpreted with caution and may represent an incidental finding.

Our study has several limitations. First, the sample size of AE patients was small, and the types of AE were heterogeneous. Our findings suggest a potential association with the development of AE, but they may not differentiate the genetic backgrounds of the various forms of AE. Collaboration among multiple centers is warranted to include a sufficient number of patients and clarify the different genetic backgrounds among various AEs. Second, we only examined deleterious variants in the exon using WES, which could miss non-coding intron regions. The non-coding intron regions can disrupt normal splicing patterns, leading to changes in protein expression levels or receptor functionality [55]. Third, we used a commercial kit with a cell-based assay to test for six known antibodies associated with AE, as tissue immunohistochemistry assays were unavailable at our facility. Consequently, some cases in our study group classified as antibody-negative may not have been genuinely antibody-negative. To avoid including non-AE patients among the probable antibody-negative AE cases, we adhered to the experts’ consensus diagnostic algorithm [3,6] and required a positive response to immunotherapy for inclusion in this study. Fourth, our analysis was restricted to germline DNA variants in the peripheral blood. We did not look for somatic mutations that may be induced by environmental factors or infectious agents and could play a direct role in the pathogenesis and onset of AE. Lastly, there is no functional analysis of how the genetic variants observed in this study participate in the pathogenesis of AE. Further functional studies of the genetic variants are required to understand their role in AE.

## 5. Conclusions

Our findings indicate that rare deleterious genetic variants in immunological genes are associated with an increased risk of developing AE. The identified RDV in our study may have a potential role in the pathogenesis of AE. As research in this field advances, we anticipate a deeper understanding of AE’s pathophysiology, driven by abnormal immunological responses.

## Figures and Tables

**Table 1:** The demographic characteristics of patients with autoimmune encephalitis.

Type of AE	Number of Patients	Gender (Female/Male)	Age (IQR)
Anti-NMDAR encephalitis	12	10/2	25.5 (21.0–32.25)
Anti-GABABR encephalitis	1	0/1	53.0
Anti-LGI1 encephalitis	1	1/0	25.0
Probable antibody-negative AE	22	13/9	35.0 (26.0–45.5)

Aberrative: AE = autoimmune encephalitis; GABABR = gamma-aminobutyric acid-B receptor; IQR = interquartile range; LGI1 = leucine-rich glioma-inactivated 1; NMDAR = N-methyl-d-aspartate receptor.

## Data Availability

The datasets generated and analyzed during the current study cannot be made openly due to ethical concerns but are available from the corresponding author upon request.

## Supplementary Materials

The following supporting information can be downloaded at: https://www.mdpi.com/article/10.3390/neurolint17120199/s1, Table S1: The diagnostic criteria for possible autoimmune encephalitis; Table S2: The diagnostic criteria for definite autoimmune limbic encephalitis; Table S3: The diagnostic criteria for anti-N-methyl-d-aspartate receptor encephalitis; Table S4: The diagnostic criteria for probable antibody-negative autoimmune encephalitis; Table S5: table for genetic variants.
