# Supplementary material for: Rare Variants of Immune-Related Genes Increase Susceptibility to Autoimmune Encephalitis: An Association Study
Source: Neurol Int. 2025 Dec 8;17(12):199. doi: 10.3390/neurolint17120199 (PMC12735519; doi:10.3390/neurolint17120199)
Supplement: Supplementary file 1 [file neurolint-17-00199-s001.zip › Supplementary Table 1-4 supplement tables for diagnostic criteria for AE.pdf]

Supplementary Table 1. The diagnostic criteria for possible autoimmune encephalitis (Graus, Titulaer et al. 2016).

Meet all three of the following criteria:

1. A subacute onset of working memory deficits, change of mentality status, or psychiatric symptoms that progressed for fewer than three months.
2. One of the following must be presented:
   - New focal neurological findings of the central nervous system.
   - New-onset seizures or seizures that can’t be attributed to a previously known seizure disorder.
   - The white blood cell count of cerebrospinal fluid is more than five cells per mm^3.
   - Encephalitis is suggested by magnetic resonance imaging.
3. Reasonable exclusion of alternative causes.

Supplementary Table 2. The diagnostic criteria for definite autoimmune limbic encephalitis (Graus, Titulaer et al. 2016).

Meet all four of the following criteria:

1. Subacute onset of working memory deficits, seizures, or psychiatric symptoms suggesting the involvement of the limbic system that progressed for fewer than three months.
2. Bilateral brain abnormalities on T2-weighted fluid-attenuated inversion recovery magnetic resonance imaging are predominantly localized to the medial temporal lobes.
3. At least one of the following:
   - The white blood cell count of cerebrospinal fluid is more than five cells per mm^3.
   - Electroencephalography reveals epileptic or slow-wave activity in the temporal lobes.
4. Reasonable exclusion of alternative causes.

Supplementary Table 3. The diagnostic criteria for anti-N-methyl-d-aspartate receptor encephalitis (Graus, Titulaer et al. 2016).

The presence of one or more of the six symptoms below with positive anti-N-methyl-d-aspartate receptor, after reasonable exclusion of other disorders.

1. Abnormal psychiatric behavior or cognitive dysfunction.
2. Speech dysfunction such as pressured speech, verbal reduction, or mutism.
3. Seizures.
4. Movement disorder including dyskinesias, rigidity, or abnormal postures.
5. Decreased level of consciousness.
6. Autonomic dysfunction or central hypoventilation.

Supplementary Table 4. The diagnostic criteria for probable antibody-negative autoimmune encephalitis (Dalmau and Graus 2023).

Meet all four of the following criteria:

1. A subacute onset of working memory deficits, altered mental status, or psychiatric symptoms that progressed for fewer than three months.
2. Exclusion of well-defined syndromes of autoimmune encephalitis (eg, limbic encephalitis, Bickerstaff's brainstem encephalitis, acute disseminated encephalomyelitis).
3. Absence of well-characterized autoantibodies in serum and cerebrospinal fluid, and at least two of the following criteria:
   - Magnetic resonance imaging suggests autoimmune encephalitis (symmetric or asymmetric).
   - Cerebrospinal fluid pleocytosis, the presence of cerebrospinal fluid-specific oligoclonal bands, or elevated cerebrospinal fluid IgG index.
   - Brain biopsy showing inflammatory infiltrates and excluding other disorders.
4. Reasonable exclusion of alternative causes

Dalmau, J. and F. Graus (2023). "Diagnostic criteria for autoimmune encephalitis: utility and pitfalls for antibody-negative disease." Lancet Neurol **22**(6): 529-540.

Graus, F., M. J. Titulaer, R. Balu, S. Benseler, C. G. Bien, T. Cellucci, I. Cortese, R. C. Dale, J. M. Gelfand, M. Geschwind, C. A. Glaser, J. Honnorat, R. Hoftberger, T. Iizuka, S. R. Irani, E. Lancaster, F. Leypoldt, H. Pruss, A. Rae-Grant, M. Reindl, M. R. Rosenfeld, K. Rostasy, A. Saiz, A. Venkatesan, A. Vincent, K. P. Wandinger, P. Waters and J. Dalmau (2016). "A clinical approach to diagnosis of autoimmune encephalitis." Lancet Neurol **15**(4): 391-404.
